# “*Existimos*”: Health and social needs of transgender men in Lima, Peru

**DOI:** 10.1371/journal.pone.0254494

**Published:** 2021-08-02

**Authors:** Sari L. Reisner, Alfonso Silva-Santisteban, Ximena Salazar, Jesse Vilela, Lynne D’Amico, Amaya Perez-Brumer

**Affiliations:** 1 The Fenway Institute, Fenway Health, Boston, Massachusetts, United States of America; 2 Division of Endocrinology, Diabetes and Hypertension, Brigham and Women’s Hospital, Boston, Massachusetts, United States of America; 3 Department of Medicine, Harvard Medical School, Boston, Massachusetts, United States of America; 4 Department of Epidemiology, Harvard T.H. Chan School of Public Health, Boston, Massachusetts, United States of America; 5 Center for Interdisciplinary Research in Sexuality, AIDS, and Society, Universidad Peruana Cayetano Heredia, Lima, Peru; 6 Sociedad Trans FTM Perú, Lima, Peru; 7 Dalla Lana School of Public Health, University of Toronto, Toronto, Canada; Oregon State University, UNITED STATES

## Abstract

**Background:**

The health of transgender men (trans men)–individuals who identify as men and were assigned a female sex assigned at birth–is overlooked globally. This mixed-methods exploratory study sought to understand the lived experiences, health, and social needs of trans men in Lima, Peru to bring visibility to specific health needs and inform responsive and holistic public health efforts.

**Methods:**

Between July 2016-January 2017, 46 trans men in Lima, Peru participated in a mixed-methods study. Four focus group discussions were conducted, complemented with 10 one-on-one interviews to explore in-depth issues that arose in groups. Two individuals participated in both a focus group and an interview. All participants completed a brief survey assessing sociodemographic characteristics and experiences with healthcare, mental health, and stigma. Audio files were transcribed verbatim and analyzed using an immersion crystallization approach to identify themes.

**Results:**

Participants had a mean age of 24 years (range 18–48). Trans men reported a lack of awareness and information among medical providers, avoidance of healthcare due to discrimination and maltreatment, an absence of public services for medical gender affirmation (hormones, surgeries), and unmet mental health needs. Trans men described health as multidimensional and influenced by social, economic, and legal contexts including family, school, employment and work, legal identity recognition, discrimination in public spaces, and peer support. Violence, stigma, and intersecting forms of oppression were described as limiting social and legal recognition of trans identity a central dimension of health. Peer support, often in an online environment, was described as important to resistance and well-being.

**Conclusions:**

Findings demonstrate that the physical and mental health of trans men, as well as unmet needs for healthcare services, are influenced by a complex set of social, economic, and legal challenges due to the social exclusion of trans people in Peruvian society. Results are a call to action for stakeholders in Peru to guarantee the rights, health, and wellbeing of this community.

## Introduction

Transgender men (trans men)–individuals who identify as men, male, or another masculine gender identity different than their female sex assigned at birth–remain an overlooked population globally, including in Peru [1]. In the urban center of Lima, Peru’s capital city, transgender people who express a gender different from the one assigned to them at birth experience social and economic exclusion, stigma and discrimination, and violence victimization at an alarming rate of occurrence, a social reality that continues to threaten the health of this historically marginalized group [2–4]. Studies of transgender women in Peru indicate complex and suboptimal interactions with healthcare systems and services, such as mistrust due to experiences of mistreatment by providers and others in healthcare (e.g., facility security personnel, other patients), barriers to receipt of medical gender affirmation (e.g., lack of clinical protocols, lack of trained providers, lack of insurance coverage) leading to medically-unmonitored hormone use and/or procedures for body modification (e.g., silicone), and obstacles due to social determinants (e.g., inability to change name/gender marker on legal documents, financial hardship due to discrimination in employment) making access to and use of healthcare services difficult [2–6].

While research in transgender health is expanding, trans men continue to be underrepresented in the global health literature [1, 7]. This erasure can be traced to the lack of HIV funding for trans men communities, the historical source and health catalyst of much transgender research, largely focused in transgender women [8–11]. A recent scoping review of research in low-income and middle-income countries about trans men identified 53 studies, only 1 of which was in Peru, and described avoidance of care due to negative experiences, stigma from healthcare providers, and barriers to gender-affirming care (e.g., lack of qualified and willing providers, high cost, restrictive gatekeeping in assessment for hormones and surgery) as common themes [7]. Trans men’s disengagement in healthcare may have serious health-related consequences including increased morbidity and mortality [12]. For example, lack of routine sexual health screening in trans men may increase risk of reproductive cancers or HIV acquisition [13]. More research is needed to holistically understand the lived experiences of transgender men across the world, including the social, economic, and legal contexts surrounding health risks and strategies for health promotion.

Although transgender people are recognized as a group of “special protection” in the National Human Rights plan [14], legal frameworks do not guarantee some of the fundamental rights of this population. There is no gender identity law in Peru, which poses a barrier for transgender people to access a legal identity that conforms to their felt and lived identity, and facilitates exclusion and discrimination. Most research describes the legal context for transgender women and shows the urgent situation regarding access to health services, education, and employment [4, 15]. There is very little systematic information from official, academic, or community sources regarding trans men.

The objective of the study is to raise visibility, document, and understand the healthcare needs and contexts shaping the health and well-being of transgender men in Lima, Peru to inform responsive public health efforts.

## Materials and methods

### Participants and procedures

Between July 2016-January 2017, 46 trans men in Lima, Peru participated in an exploratory mixed-methods study to assess and comprehend their health and social situation, in the context of their lived experiences, socialization dynamics, and access to public services.

The study was conducted by the UNICXS project, an anti-violence reporting and response project focused on transgender health and human rights in Lima, Peru. Trans men were purposively sampled and recruited through community-based methods and partnerships with local community members and groups. A transgender man peer recruiter posted study flyers and invited individuals to participate in the study through in-person or online outreach, such as one-on-one at community events or through the posting and publication of a study flyer in a Facebook group with trans men community members. Inclusion criteria for study participation were: (1) ages 18 years or older; (2) female sex assigned at birth who feel and/or express a masculine gender identity, regardless of any process of bodily transformation or medical gender affirmation; (3) reside in Lima at the time of the study; and (4) able to give informed consent.

Data collection sources were in-depth interviews, focus group discussions, and a brief survey. Four focus group discussions with 8 to 10 participants each were conducted (n = 38 total trans men), complemented with 10 one-on-one interviews (n = 10 trans men) to explore in-depth issues that arose from focus groups. Two individuals participated in both a focus group and interview (total unduplicated participants N = 46 trans men). The interviews and focus groups were held at offices at Centro de Investigación Interdisciplinaria en Sexualidad Sida Y Sociedad (CISSS-UPCH), at the Universidad Peruana Cayetano Heredia (UPCH).

Participants received compensation equivalent to US $10 for time taken to participate on focus groups and surveys. The protocol was reviewed and approved by the Institutional Ethics Committee of UPCH in Lima, Peru (No. 66604) and The Fenway Institute at Fenway Health in Boston, MA USA (No. 771415). All participants provided oral informed consent to the researchers who conducted focus groups and interviews. Only the researchers and the transcribers had access to the audio and text transcription of focus group discussions and interviews.

### Measures

Three sources of data were collected. Focus groups and interviews utilized semi-structured qualitative guides which covered the following areas: socialization (family, friends, intimate partners), access to education, work, health, experiences of discrimination and violence, social and community support, and life expectancy. Focus groups and interviews were audio recorded and transcribed verbatim. Any confidential or personal information that could potentially identify participants was removed.

At the time of the focus group discussions and interviews, participants were also asked to complete a brief survey. To better understand participants’ sociodemographic characteristics, the survey assessed: (age, student status, income, housing); gender identity (man, male, transgender man, genderqueer); gender development milestones (first age of transgender awareness, disclosure of transgender identity); gender affirmation (current use of and access to hormones and surgeries); healthcare experiences and stigma in healthcare (4-items); mental health (PHQ-2 screener); and emotional and social support (4-items).

### Data analysis

Focus groups and interviews were analyzed using an immersion crystallization approach [16, 17] to identify themes and relationships between themes. Applying this qualitative approach, data collection and analysis were implemented as a reciprocal process where each informed the other as well as the subsequent steps in data analysis and dissemination. This approach emphasized both examining data and reflecting on the experience of being immersed in data analysis. Transcripts were analyzed based on an inductive and deductive approach to identify codes and relationships between codes in iterative development of the codebook. Transcripts were analyzed using Dedoose Version 6.1.18 (2015) for qualitative analysis, using a list of basic codes and sub-codes. The first step of the analysis was coding of the transcripts. The codes and sub-codes generated by the research team were sorted into a coding template after analyzing the transcription of a first focus group. The codes and sub-codes generated in the template were agreed upon by the research team and were then applied to the other transcripts. Codes and sub-codes were organized into a matrix to identify specific topics. Finally, relationships were established between each of the topics to achieve a finer interpretation of the information collected. The qualitative analysis was complemented with sociodemographic and health information obtained through the survey. Qualitative results were thematically and not numerically summarized; thus, all numerical data reported (i.e., percentages) are from the quantitative survey and are meant to complement, support, and enhance qualitative findings. Surveys were analyzed by describing simple frequencies of variables using STATA software version 13.0.

## Results

### Study sample

#### Sample characteristics

Characteristics of the study sample are presented in Table 1. Participants had a mean age of 24 years (range ages 18–48 years), reflecting a sample that was mostly young; 42% were current students, and most frequently reported their sources of primary income as 58% employment (full- or part-time) and 21% parents. The majority (87%) lived with family/extended family or 16% partner/boyfriend/girlfriend/spouse. Most participants reported residing in the *Centro de Lima* (central district of Lima).

**Table 1:** Characteristics of the study sample of trans men in Lima, Peru (n = 46).

Age in years	**Mean**	**SD**
Range 18 to 48 years	24	5.3
	**N**	**%**
Currently a student		
No	21	55.3
Yes	16	42.1
No Response	1	2.6
Primary source of income		
Employment (Part or Full Time)	22	57.9
Parents	8	21.1
No Income	2	5.3
No Response	5	13.2
Other (Tips, Rent from Tenants)	2	5.3
How many people live in participant’s home:		
2 to 5 people	25	65.8
6 to 10 people	11	28.9
More than 10 people	2	5.3
What is the relation of housemates to participant:		
Friends	2	5.3
Family/extended family	33	86.8
Partner/boyfriend/girlfriend/spouse	6	15.8
Tenants	2	5.3
Cousins	1	2.6
Sex assigned at Birth		
Female	38	100.0
Gender Identity		
Male/ Man	33	86.8
Transgender Man	18	47.4
Transmasculine	3	7.9
Masculine	1	2.6
At what age did you FIRST become aware that you were transgender or gender nonconforming?	**Mean**	**SD**
Range 3.5 to 30 years	12	6.9
*Write in*:	**N**	**%**
"Forever"/"For as long as I can remember"/"Since I had the use of reason"	4	10.5
At what age did you first tell another person that you are transgender/gender nonconforming?		
Range 8 to 30 years	18	4.4
Who have you told that you are transgender/gender nonconforming?		
Friends	36	94.7
Partner	30	78.9
Housemates	14	36.8
Classmates	19	50.0
Work colleagues	13	34.2
Healthcare provider	4	10.5
Siblings	28	73.7
Parents	26	68.4
Children	1	2.6
Extended Family	21	55.3
Others	4	10.5

#### Gender identity and transgender identity development and disclosure

As shown in Table 1, trans men most commonly described their gender identity as 87% male/ man, 47% transgender man, and 8% transmasculine. Varying rates of transgender identity disclosure were reported and depended on who they were disclosing to (95% friends, 79% partner, 74% siblings, 69% parents). Participants reported first becoming aware they were transgender at a mean age of 12 (range ages 3–30 years). Four participants wrote-in a response: “*forever*”, “*for as long as I can remember*”, and “*since I had the use of reason*”. Trans men reported first telling another person about being transgender at a mean age of 18 years (range ages 8–30 years).

### Healthcare experiences and unmet health needs

Themes regarding healthcare experiences and unmet health needs of participants coalesced around six primary topics: (1) Avoidance and limited use of healthcare; (2) discrimination in healthcare; (3) Lack of awareness and information among medical providers; (4) Unmet medical gender affirmation needs; (5) Sexual and reproductive health needs; and (6) Mental health needs.

#### Avoidance and limited use of healthcare

Participants described avoidance and limited use of healthcare due to previous experiences of maltreatment and associated high levels of mistrust toward health providers and the medical establishment. All participants (100%) reported discomfort with healthcare providers as a deterrent to accessing health services. More than half (55%) reported not feeling comfortable interacting with health personnel or talking about their gender identity with them (53%). Almost three quarters (72%) reported not trusting that providers could help them with medical gender affirmation processes. Only 11% reported having ever disclosed they were transgender to a healthcare provider. One participant described how the non-recognition of his gender identity on his National Identity Document (DNI, *Document Nacional de Identidad*) and discrimination experiences deterred him from accessing healthcare:

“*Well*, *I´ve never liked going to the hospital because of those issues*, *the ID (DNI)*, *the name your appearance and that stuff*, *right*? *I mean*, *I´ve been in the hospital and seen physicians or nurses making gestures or saying things that don´t make me feel good*. *So I´ve never liked seen a doctor*”

(FG #1).

#### Discrimination in healthcare

Overall, 69% of participants reported experiencing discrimination or mistreatment from health personnel in relation to their gender identity. One participant recounted his experience:

“*Yes*, *then I went in and left*, *and they kept looking at me weird*. *Well*, *the doctor up to then (was) super polite and everything*, *but when they were going to check me*, *the nurse came and started talking*: *‘Is she a woman or a man*?*’ and I (was) super uncomfortable*, *and until now I have an abscess there and I have not been (back) to be seen*.”

(FG #2)

In addition to discrimination from healthcare personnel, participants reported experiences of discrimination and mistreatment from others in the healthcare environment, including from front desk and administrative staff (e.g., receptionists), security personnel, and fellow patients.

A common barrier that participants described to accessing care (and to accessing any official institution) was the need to present a DNI. Participants discussed how their DNI reflected the sex assigned at birth, drawing attention to the discrepancy between their current gender identity and assigned sex at birth. As one participant described: “*I have never liked going to the hospital or a doctor because of my DNI… my name and my appearance*.” (FG #1)

#### Lack of awareness and information among medical providers

Participants reported a widespread lack of awareness and information regarding transgender identity and health needs among medical providers. One participant narrated an experience he had with an uninformed provider this way:

“*Of course*, *I had already made my transition*, *I had my hair cut*, *I went with my mother*, *I remember and the doctor checked me*. *I had not menstruated for about 4 months and I was worried*. *The doctor took my mom out of the office and told me hija (daughter) you are pregnant*. *I told him that was impossible*. *What happened was that I had a tumor on my right ovary that I had to have removed and the tumor was so big it was the size of a ball*. *The doctor*, *like he did not have much understanding at the time (about transgender issues)*, *did not have the required education in the subject of trans men*.”

(FG #1)

Frequently trans men described the lack of clinical training and protocols in Peru for providing healthcare to transgender people, especially for trans men, as an unmet health need. Further, participants described that while transgender women had some visibility in Peruvian society and within medical institutions, trans men were erased. As described, “*I would like to society to recognize us*. *We exist*. *We are people*. *We are not one*, *we are many… We need respect we need to our identity*.” (Interview #6)

#### Unmet medical gender affirmation needs

Nearly all participants described gender affirmation as a health priority. Medical gender affirmation characteristics of the sample are presented in Table 2. Participants most commonly expressed a desire for hormone therapy and chest reconstruction. However, they indicated that services for hormone therapy were difficult to locate, non-existent within public institutions, and very scarce and costly in the private sector. For example, the cost of hormones, around 200 soles per month ($60), was reported to be unaffordable for many participants.

**Table 2:** Medical gender affirmation in the study sample of trans men in Lima, Peru (n = 46).

	N	%
Have you ever taken male/masculinizing hormones (e.g., testosterone)?		
Yes	12	31.6
No	26	68.4
What form(s) of testosterone have you ever used? Check all that apply.		
Intramuscular injection	12	100.0
Gel, cream, or patch	1	8.3
Oral/pill	1	8.3
Where have you gotten your hormones? Please check all that apply.		
From a clinic or health center	4	33.3
From a pharmacy	10	83.3
From a private practice	1	8.3
Would you like to use male/masculinizing hormones (e.g., testosterone) at some point in the future?		
Yes	33	94.3
No	1	2.9
I don’t know	1	2.9
Have you ever undergone gender affirming surgery?		
Yes	1	2.6
No	37	97.4
If yes, what kind?		
Top surgery	1	2.6
Would you like to have gender affirming surgery in the future?		
No	2	5.3
Yes	34	89.5
Don’t Know	1	2.6
No Response	1	2.6
*Write in options specified*:		
Mastectomy/Top Surgery	10	26.3
Phalloplasty	2	5.3

Participants reported accessing information about hormone therapy through the Internet or peers. Approximately 30% reported having used male hormones, although the majority of participants (94%) reported wanting to use them. Of those using hormones, all reported using intramuscular injection. Pharmacy (83%), clinic or health center (33%), or private practice (8%) were the most commonly reported procurement sources for hormones.

Participants described the absence of public health services for medical gender affirmation and the importance of making medical gender affirmation accessible, particularly in the context of societal stigma:

“*It’s important because if you physically look like a man*, *people will think you are a man*, *and they will treat as such*. *The problem if you still have feminine characteristics people will see you and think ‘here something is weird’*.”

(FG #4)

Information regarding access to surgical procedures for trans men was described as rare relative to access to hormone therapy. Participants described both the high costs of surgery and the limited number of providers offering transgender surgical interventions as key barriers to accessing these types of gender-affirming services. The procedure participants mentioned most was chest surgery (e.g., mastectomy, chest reconstruction). Yet, only one participant reported having undergone chest surgery, although more than 90% reported the desire to have that type of surgery.

Due to unavailable medical gender affirmation policies and procedures within in public health institutions, participants described primarily accessing behavioral gender affirmation practices, such as, relying on binding, using constrictive materials to flatten the breast tissue and create a male-appearing chest, and packing, using a non-flesh penis or other material to give the appearance of having penis. Participants expressed a need for early gender-affirming interventions, wanting publicly available services for trans men in late adolescence.

#### Sexual and reproductive health needs

Participants did not describe gynecological care as a health need. When asked if they had ever been screened for cervical cancer (e.g., had a Pap test), the overwhelming majority said they had not. As one participant reported: “*I have never gone*, *ever*. *I’m 24 and I haven’t stepped into a gynecologist’s office*. *Not as a man*, *not as a woman*. *Ever*.” (FG #1)

Anticipated stigma and associated anxiety deterred participants from accessing gynecological care, even when needed and/or among those who had accessed care reported evading follow-up care. Participants note the difficulty in seeking this type of “female” care as a man, which they viewed as exacerbated by the absence of trained healthcare providers:

“… *I haven´t gone because I don´t know how*, *because I know it would be a problem*. *First*, *by saying your [legal] name and why you’re going to a gynecologist*, *if you look like a man; then*, *with the gynecologist*. *What am I going to say*? *Like*, *there is no one who is*, *let’s say*, *trained on these topics*, *that knows how to treat a trans person in a way that doesn’t make them feel all that anxiety when going to a doctor*. *That’s really needed*.”

(FG #1)

To alleviate gender policing and stigma in communal waiting rooms, some participants described strategies they had used when accessing gynecological care. For example, one strategy was going with their female partners, sisters, or their mothers to avoid situational discomfort with front desk staff, nurses, and providers. As one participant reported:

“*I took advantage when I was in a relationship to go and get checked together*. *then [my girlfriend] would say to me ’we are going to get checked*. *We are not going so that they will also check you’*, *and I would go and talked to the doctor*. *I would always look for a female doctor*, *for different reasons*, *and then we both got checked*. *But when I don’t have a girlfriend*, *I don’t go*.”

(FG #2)

#### Mental health needs

Daily experiences of psychological distress and anxiety were reported by all participants. Many described struggling with self-acceptance and reported feelings of self-blame, guilt, and shame about being transgender. One participant linked his transgender identity to feelings of guilt: “*And then I said ‘I have something*, *something is going on with me*, *I mean*, *I don’t have to be like that [trans]*,*’ I mean*, *I felt guilty*, *right*?” (FG #4) In many cases, participants reported social isolation and feeling alone, as if they were the only person in the world who was transgender.

Participants commonly described the internal conflict resulting from the clash between their felt gender identity as men and the social expectations and gender norms associated with their female sex assigned at birth. One participant described experiencing this as a betrayal of his transgender identity because he felt he had been living a lie: “*I felt like Pinocchio*, *I could never truly become myself*, *I felt as though I was always going to stay this way [in between two genders]*.” (Interview #1)

Several participants reported suicidal ideation and attempts associated with feelings of loneliness. One participant described social isolation due to bullying he experienced based on not conforming to gender norms in childhood and highlighted the toll this took on his mental health:

“*I was isolated and ridiculed…*. *I only went between school and my room*. *As a result*, *I was super depressed and tried to kill myself two times*.”

(Interview #1)

Participants described the lack of available mental health services, particularly the lack of publicly funded mental health services. Participants relayed difficulties finding mental health providers who were knowledgeable about the mental health concerns of transgender people and trained in working with trans men specifically.

### Social, economic, and legal contexts

Social, economic, and legal contexts emerged as important situational factors in the health and healthcare of trans men. Themes coalesced around seven primary topics: (1) family, (2) school, (3), employment, (4) legal identity recognition, (5) discrimination in public spaces, (6) societal conflation of sexual orientation and gender identity, and (7) peer support. Everyday stressors experienced in relation to participants’ gender identities within these domains were described as sources of anguish detrimental to their health and well-being.

#### Family

Participants commonly described rigid and conservative gender norms in their family growing up as confining and stifling. They described frustration, anger, and ultimately sadness at not being able to express their masculinity visibly and outwardly in childhood, and the long-term negative mental health effects of this hiding. The act of “coming out” was recounted by many participants as an abrupt turning point in their lives. For most, the first disclosure of their transgender identity was to family members, in particular to their mothers. One participant shared:

“*I felt I just couldn’t go on and I told my mother everything…My hair had been long since I had been 14 and I said*, *‘No*, *I just can’t take any more’ and I grabbed and one day I left the house*, *I went to a party and got drunk and cut my hair [laughs]*. *And like that I went home*, *and my mother started to cry*.”

(FG #2)

Participants narrated variable experiences with their families and familial acceptance of their being transgender. While some reported maintaining relationships with their family, others were no longer able to. Participants with conservative or religious families described the greatest family difficulties. Even in families that were accepting, participants reported common struggles, particularly the harms associated with being misgendered: “*what is most difficult to change are the names when of calling us because*, *it’s been like*, *twenty years that they have called you one way*, *and to change overnight*, *I understand it can be confusing for them*” (FG#1)

#### School

Participants described educational institutions as challenging due to experiences of gender policing related to the sex-segregated dress code at school, as well as moments of resisting these: “*My mom sent with me my nice little hairdo*, *with my little skirt*, *and in the middle of the street I took everything off and put on my track pants*. *-Yeah*, *I also had my track pants in my backpack*.” (FG #4) Participants described how transgressing gender norms in school was generally not accepted by peers or school faculty and resulted in harassment and bullying victimization. Participants explained how others refer to them using pejorative terms such as “*bicho raro*” (literally, *strange animal*) (FG #4) and “*la rara*” (the *strange one*) (Interview #8). Others recounted harassment and bullying experiences beyond name-calling:

“*They (my peers) would take photos of me on their cell phones while I was in the bathroom*. *They would mock me and talk about me as though I wasn’t there*. *I was isolated*, *never included*.”

(Interview #10)

Participants explained that schools have little or no information on issues related to gender identity or gender diversity. Participants also reported varying experiences of acceptance or rejection and discrimination at the institutional level according to the type of school attended. Numerous participants reported attending a religious school and described how religious beliefs and teachings on “homosexuality as sin” fueled rejection by family and peers, as well as inhibited their own self-acceptance. As one participant described:

“*I went to an evangelical school and in those years…We were taught that homosexuality was a sin and that homosexuals would go to hell*, *and at that age I believed it*. *Psychologically*, *I couldn’t accept myself because I thought I would go to hell*… *I tried to remind myself that I wanted God to love me and forced myself to dress like a woman and to try and forget how I felt*.”

(Interview #1).

Trans men often reported that finishing school marked the turning point in their lives when they openly began to transition and express their “*true selves*.” However, participants reported that the challenging experiences they had in school settings remained with them, including resultant feelings of internalized shame and poor mental health.

#### Employment

Participant narratives about employment were varied and differ according to job type. Approximately 70% reported some form of employment, yet participants pointed to difficulties finding employment. One participant described his challenges:

“*…but unfortunately in questions of being able to apply my capabilities*, *I have applied*, *I have (submitted) more than 105 applications…they do not give me the option*. *And there are people who*, *I do not want to say that they are bad either*, *but (who are less capable)…they enter positions that I have been seeking for 3 years…*”

(FG #4)

Participants also discussed difficulties of precarious employment: “*I was working cleaning offices and they got rid of me in a month for being like this (trans)*” (FG #2).

Trans men reported ongoing complications carrying out their workplace responsibilities because of their transgender identity. These challenges included threats of being “outed”, having their identity questioned, and being discriminated against because of it. Participants commonly alluded to limitations accessing and exercising their right to work due to the incongruence between their gender identity and their legal identity. Participants described how they were misrepresented on the DNI, which legally designates sex assigned at birth rather than current gender identity, and the ongoing difficulties that caused for employment.

#### Legal identity recognition

All participants reported difficulty changing their name and gender markers on official forms of identification. They highlighted this lack of legal recognition as a chronic stressor affecting their health and as a structural barrier to everyday living. Participants viewed the DNI as the primary barrier to accessing healthcare services, employment, and other resources (e.g., banking, credit). All participants associated discrimination, fear, mental health distress and anxiety, and social exclusion with the need to present a DNI which was not concordant with their gender identity.

“*It´s always tedious to do any procedure that involves the DNI*.”

(FG#3)

“*they call you by your name and stare at you like a weirdo (bicho raro)*, *and they you- it´s that you*? *And you blush because its embarrasing*, *right*?”

(FG#1)

#### Discrimination in public spaces

Trans men reported widespread experiences of discrimination, often in public spaces (e.g., public restrooms), including in healthcare settings. These experiences were often attributed to their physical appearance (e.g., inability to “pass” as male) and/or to their transgression of gender norms (e.g., presenting as masculine). Participants reported public restroom use as an unavoidable and challenging situational context in their daily lives. As one participant described:

“*I always have issues entering a bathroom*. *Honestly*, *I am scared to enter a public bathroom*. *Because in the women’s bathroom*, *I am not enough of a woman to enter and for the men’s bathroom*, *I am not masculine enough*.”

(FG #4)

Participants perceived that medical gender affirmation (e.g., hormones) was not only important to their self-perception and self-actualization as men, but also necessary for easier day-to-day navigation of public spaces such as restrooms and public healthcare settings.

#### Conflation of sexual orientation and gender identity in Peruvian society

Many participants reported being unaware of the concept of transgender identity until the late stages of adolescence or adulthood. However, they often described in their narratives an early identification with the masculine through their choices of clothing, toys, or identification with a male close to them such as a sibling or father. Participants recounted common questions of ‘who am I’ and ‘what am I’ when searching for “*[their] true self*” (“*mi verdadero yo*”, Interview #6) in late adolescence or young adulthood. They highlighted the need for social support during that process.

Trans men commonly recalled perceiving and naming themselves as butch lesbians (“*activa*”, “*chito*”) and confusing gender identity with sexual orientation. Participants emphasized the need for greater societal awareness of and access to information about the distinction between sexual orientation and gender identity and expression. Further, they described an absence of role models as transgender boys, and lack of cultural references (“invisibility”) in society as transgender men.

“*And when I supposedly came out of the closet*, *I didn’t have the necessary information*, *and because I liked girls and I was woman*, *then I’m a lesbian*, *right*? *What other option did I have if I didn’t know about the topic*.”

(FG#2)

#### Peer support

Trans men reported resistance and self-care strategies rooted in peer social support, often obtained virtually online. Participants frequently described Facebook, chats, and other online platforms as crucial to circumnavigating feelings of isolation, learning about trans men identity, and understanding medical gender affirmation needs. One participant conveyed his experience:

“*I started to find out about the meaning of the word [trans]*. *I started to look on the Internet and I began to see that there were other people*. *I started to see a group of more people who were trans and I felt identified and I said I am like that*. *And as I felt identified somewhere and started to find out*, *I started to inform myself*, *I found other trans people through the internet*, *who guided me and helped me*.”

(FG #3)

Participants described the Internet as a resource for health, and a safe space to access needed information and seek social support. The Internet was described as enabling them to understand they were not alone and that *trans manhood* existed. They reported how the Internet was instrumental to becoming aware of peers, especially others living in their city, and how it boosted self-acceptance of their own transgender identity.

## Discussion

This study presents a first-hand perspective of the health and healthcare needs of trans men in Lima, Peru. Trans men exist and narratives described herein highlight the ways that the invisibility of trans men within healthcare and public health contexts adversely impact their health and well-being. Several unmet healthcare needs were identified in this study, particularly those pertaining to lack of publicly available medical gender affirmation interventions and mental healthcare services. Findings demonstrate that the physical and mental health of trans men, as well as their unmet needs for healthcare services, are influenced by a complex set of social, economic, and legal challenges due to the social exclusion of transgender people in Peruvian society [18]. Multilevel systems of oppression (i.e., stigma, policing) and limited social recognition of transgender identity emerged as predominant contexts in which to understand the health and healthcare needs of this underserved population [19]. Social exclusion of trans men in Peru is driven by the absence of a legal framework and systemic reinforcement of binary gender categories linked to biological sex across varied contexts, including in the healthcare, family, school, employment, and legal realms [20]. Results from this study represent a call to action for stakeholders in Peru to guarantee the rights, health, and wellbeing of trans men.

Corroborating research from other parts of the world [21–23], this study found that both actual and anticipated experiences of discrimination in healthcare settings were all too common for trans men in Peru. The primary barrier to healthcare access for trans men was the DNI and having a gender presentation not matching legal identification. This structural barrier fundamentally limited access to healthcare resources for trans men and represents a basic violation of human rights. Prior research has shown that legal gender affirmation is associated with improved health in trans people, including lower reports of depression, anxiety, somatization, and global psychiatric distress [24], and lower prevalence of suicidal ideation [25]. In this study, lack of legal recognition was stress-generating for participants given the structural hurdles it created across multiple domains (e.g., healthcare, work), and contributed to poor health for trans men, particularly poor mental health and psychosocial functioning.

Consistent with prior research [1, 7], trans men in this study reported avoidance of healthcare and limited use of health services. Rather than exposing themselves to discriminatory treatment in healthcare settings, uninformed or misinformed healthcare providers, and potential ridicule and humiliation from others (e.g., healthcare administrators, fellow patients), trans men reported disengagement from healthcare, a finding which corroborates prior research globally [7, 23]. Avoidance of healthcare poses a critical threat to the health of trans men. For example, avoiding routine preventive healthcare may enhance the risk for reproductive cancers typically detected through early screenings (e.g., cervical, breast). It may also impact HIV acquisition for trans men engaging in HIV risk behaviors who would benefit from biomedical HIV prevention interventions such as behavioral risk reduction counseling and pre-exposure prophylaxis (PrEP) care. Indeed, this study found formidable barriers to sexual and reproductive health services for trans men in Peru. Further, trans men participants on hormones did not report any discussion of fertility preservation [26] or reproductive options. The anxiety of gynecological visits for trans men in this study was fueled by fear of mistreatment, absence of trained healthcare providers, and participants’ own discomfort with obtaining care pertaining to their birth-assigned anatomy. Trans men identified the absence of clinical and medical protocols about how to provide gender-affirming care to trans people [21] as a contributor to the lack of awareness and information about trans men among clinicians and healthcare providers.

Medical gender affirmation emerged as a high-priority area of unmet need for trans men in this study. Prior research has demonstrated the positive health impacts of medical gender affirmation, including improved mental health and psychosocial functioning [27–29]. Participants reported that lack of publicly funded services for hormone treatments and surgery in Peru was a barrier to their self-actualization. Trans men felt that access to gender-affirming medical procedures, particularly hormones and chest surgery, would help them to outwardly present in ways aligning with their gender identity rather than sex assigned at birth. They identified that having access to these interventions would likely reduce the chronic anxiety and fears they reported as a result of having to negotiate gender role tensions in everyday life (e.g., using public restrooms). Most trans men in this study reported paying for hormones out-of-pocket, a substantial financial burden. Further, participants reported that clinicians and healthcare providers lacked awareness and information about medical gender affirmation for trans men, and described a gap in clinical and medical protocols to deliver hormones and surgical interventions for transgender people in Peru.

Trans men in the current study commonly reported experiences of depression, suicidal ideation, anxiety, guilt, fear, isolation, and shame, consistent with research demonstrating high rates of mental health morbidity for transgender people globally [1, 7]. Gender socialization [30] and transgression of gender norms were strong determinants of mental health and wellbeing for trans men in this study. Within a conservative and religious society, participants described how adverse gender-related experiences in childhood and adolescence across family (e.g., parental rejection and disapproval) and school (e.g., gender-segregated dress code, bullying victimization) contexts had lasting negative effects on their mental health and wellbeing into adulthood. These findings reveal an essential need for early interventions in childhood and adolescence aimed at supporting Peruvian youth who sense from an early age a gender identity or expression different than their sex assigned at birth. Early interventions can help gender diverse children feel accepted by others and themselves, as well as provide developmentally-tailored strategies for coping with stressors associated with transgressing gender norms [31]. Family interventions for parents and other family members and school interventions for educators and administrators are also warranted. These interventions can additionally help advance less rigid views about gender in society at large, which is likely to boost the social inclusion of gender diverse children, who grow up to become trans men, in families, workplaces, healthcare services, and community activities.

Trans men described how social entities and institutions punitively reinforce traditional gender norms and expectations through stigma and social exclusion, and highlighted healthcare settings and publicly funded services, work or employment contexts, and public spaces (e.g., restrooms) as constant sources of stress and strain in their lives. These findings are aligned with structural developmental life course frameworks which theorize that poor health results from chronic and cumulative stress and strain across the lifespan due to occupying a stigmatized or marginalized position in society [32–34]. Further, participant narratives demonstrated that trans men in Peru do not seek mental health support and treatment for depression and anxiety, which can be disabling even when symptoms are mild. Findings from this study suggest that publicly funded mental health services and clinicians that are appropriately trained, knowledgeable, and competent in serving trans men are urgent mental health needs for this population.

Several limitations need to be considered in interpreting study findings. The study population was young, educated, and largely employed at least part-time. We cannot be sure if this demographic is specific to the methods utilized to recruit the sample, such as through community groups, networks, and online social media, or indicative of the trans men population in Lima. This study is exploratory and has a small sample size, though saturation was reached in focus group discussions and interviews. This work is intended as a first step. We recognize the value of considering a larger and more diverse study sample, including for a future quantitative needs assessment study. Given that our study inclusion criteria were broad in defining trans men, study findings were not stratified nor explicitly analyzed for gender diversity. Thus, we did not explore the degree to which study participants’ personal expression of gender conformed or did not conform to social norms of gender in Lima, Peru, or whether they “passed” (were “read” by others) as male or trans. Future research with larger samples is recommended to allow for comparisons of health, social characteristics, and needs of trans men by gender diversity and conformity or nonconformity to culturally-specific gender norms in Lima.

Gathering data on the health and social needs of populations is vital to inform public health efforts to ensure the wellness of underserved populations. Results highlight the range of social issues impacting the health and wellbeing of transgender men. Though further assessment of social determinants of health and social resistance and resiliency strategies adopted by trans men in Lima is still needed, results of this study should be taken as a call to action for stakeholders in Peru to guarantee the rights and wellbeing of this community.

These results underscore the critical gap in basic knowledge about transgender identity and expression, the unmet health needs of trans men, the need for knowledge and sensitivity training for healthcare providers, and the lack of existing protocols specific to providing competent and compassionate care to them. Transgender health in Peru needs to be addressed as a right in and of itself. To date, the only existing public services offering hormones to transgender people are for transgender women as a way to increase access to HIV prevention services [35]. As transgender men are not considered a “key population” in the HIV epidemic, they have not been included in the protocols for hormone treatment.

As described by participants, the lack of legal protections and rights for trans men in Peru play a critical role in shaping and enabling a social environment which poses multi-layered threats to the mental and physical health of trans men. Most notably, the lack of right to legally change gender designation on the birth certificate to reflect felt gender identity, lack of rights and protections regarding public restroom use, and lack of access to gender-affirming procedures (hormone treatment and chest surgery) emerged in the findings as critical barriers to the social inclusion of trans men in Peru. These issues can be eliminated by rights-forward changes in the law [36]. Advancing legal protections and rights related to these and other barriers to social inclusion would likely mitigate the chronic stressors of fear, anxiety, distress, and social isolation that are associated with them for trans men.
